# An Acromegaly Disease Zebrafish Model Reveals Decline in Body Stem Cell Number along with Signs of Premature Aging

**DOI:** 10.3390/biology9060120

**Published:** 2020-06-07

**Authors:** Abdalla Elbialy, Yoji Igarashi, Shuichi Asakawa, Shugo Watabe, Shigeharu Kinoshita

**Affiliations:** 1 Laboratory of Aquatic Molecular Biology and Biotechnology, Graduate School of Agricultural and Life Sciences, The University of Tokyo, Tokyo 113-8654, Japan; abdallakhiry@gmail.com (A.E.); aiga@mail.ecc.u-tokyo.ac.jp (Y.I.); asakawa@mail.ecc.u-tokyo.ac.jp (S.A.); 2 Laboratory of Fish Diseases, Faculty of Veterinary Medicine, Damanhour University, Damanhour 22511, Egypt; 3 School of Marine Biosciences, Kitasato University, Minami, Sagamihara, Kanagawa 252-0313, Japan; swatabe@kitasato-u.ac.jp

**Keywords:** acromegaly, growth hormone, aging, stem cell, zebrafish

## Abstract

In our previous publication, it was shown that growth hormone (GH) excess in acromegaly affects the cell integrity of somatic cells through increased DNA damage throughout the body and impaired DNA repair pathways. Acromegaly is a hormone disorder pathological condition that develops as a result of growth hormone over-secretion from the pituitary gland. We produced a zebrafish acromegaly model to gain a better understanding of the excess GH effects at the cellular level. Here we show that the acromegaly zebrafish model progressively reduced the number of stem cells in different organs and increased oxidative stress in stem cells. Importantly, the decline in the stem cells was even more apparent than in aged fish. The controversy and debate over the use of GH as an anti-aging therapy have been going on for several years. In this study, excess GH induced aging signs such as increased senescence-associated (SA)-β-galactosidase staining of abdominal skin and similarity of the pattern of gene expression between aged and acromegaly zebrafish. Thus, this study highlights the role of excess GH in acromegaly stem cells.

## 1. Introduction

In early life, the blood level of growth hormone (GH) is high, corresponding to rapid somatic growth. Its level gradually declines during adult life and aging. This age-related decline in plasma GH level is termed “somatopause”, which has been well documented in various mammalian species [1,2]. For a long time, age-related symptoms such as muscle mass reduction have been associated with somatopause; thus, GH therapy has been used as an antiaging drug. Early studies showed that GH treatment of men over 65 years of age significantly increased muscle mass, increased bone mineral density, and reduced adiposity [3]. Such findings do not suggest, however, that GH administration improves longevity. On the other hand, somatotropic axis mutations have been associated with longevity in mice and humans. Genetic disruption of the GH receptor gene drastically increased longevity from 25% to over 60% [4]. In humans, GH deficiency syndrome (Laron dwarfism) is associated with a significant reduction in pro-aging signaling, cancer, and diabetes [5].

Acromegaly is a hormonal disorder pathological condition that develops due to growth hormone over-secretion from the pituitary gland, predominantly by a pituitary adenoma. Although, to date, according to our knowledge, there has been no direct correlation between acromegaly and aging, some studies have shown age-related signs in acromegaly patients. Excess GH in patients with acromegaly impairs cognitive function, functional mobility, and causes muscular weakness [6]. In addition, the likelihood of using GH as an antiaging medication has been reduced because acromegaly patients with excess GH do not live long.

Studying tissue senescence, aging signs, and the impact of excess GH on acromegaly body stem cells could provide clear evidence of premature aging in acromegaly and help resolve this debate.

Oxidative stress, an imbalance between free radicals and antioxidants, and the resulting cellular damage were widely thought to play important roles in aging and various age-related diseases [7,8]. Aging is the gradual loss of tissue and organ function over time. The oxidative stress theory of aging is based on the structural oxidative damage of cellular components caused by ROS (reactive oxygen species) accumulation [9]. In addition, recently, oxidative stress was shown to play a vital role in the self-renewal of stem cells, thus affecting aging. Its activation results in a loss of stem cell self-renewal, the exit of the satellite (muscular stem cells) [10], hematopoietic stem cells (HSCs) [11], and neural stem cells [12] from quiescence, and the induction of differentiation.

Patients with acromegaly display a range of clinical symptoms and comorbidities, including hyperhidrosis, gastrointestinal illness, arthritis, carpal tunnel syndrome, fatigue, colon polyps, reproductive and metabolic disorders, neuropathy, and cardiovascular disease [13,14]. A better understanding of the consequences of excess GH in acromegaly and the identification of disturbed pathways may help to identify potential targets for therapeutic interventions. In a previous publication, we showed that excess GH in acromegaly affects the cellular integrity of somatic cells through increased DNA damage and impaired DNA repair pathways [15]. Here, our research focused on investigating the effect of excess GH on stem cells and whether the excess GH causes stem cell oxidative stress and signs of aging in the zebrafish acromegaly model.

## 2. Results

### 2.1. The Acromegaly Model Declines Body Stem Cell Numbers in Various Organs

We produced a zebrafish acromegaly model by an overexpression of tilapia GH [15]. To investigate whether excess GH influenced stem cell self-renewal, we quantified the number of stem cells as a percent of the total number of cells using a Fluorescence-activated cell sorting (FACS) analysis by measuring the fraction of SP (side population) cells, as is mentioned in Materials and Methods. Although the SP phenotype has frequently been used for the identification of adult stem cells in various human and mouse organs [16,17,18], until now, it has only been used in zebrafish for the isolation of hematopoietic stem cells (HSCs) [18], so we firstly focused on the quantification of the HSCs. The flow cytometry gating strategy used for the isolation of SP stem cells is shown in Figure S1 and was based on the stem cells efflux of Hoechst 33342 dye by ATP-binding cassette (ABC) transporter activity [19].

To verify that the SP stem cell had been isolated by the FACS, verapamil was used with the Hoechst 33342 stain as a negative control because it is an inhibitor of ABC transporters. Thus, using verapamil diminishes the SP phenotype [20].

**Figure 1:**
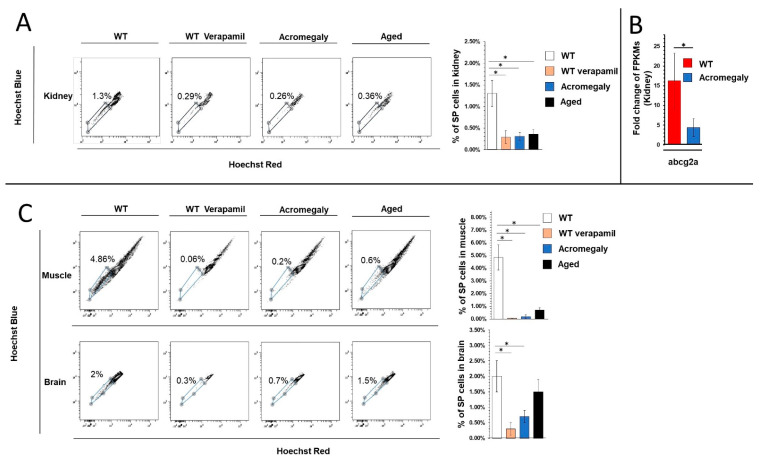

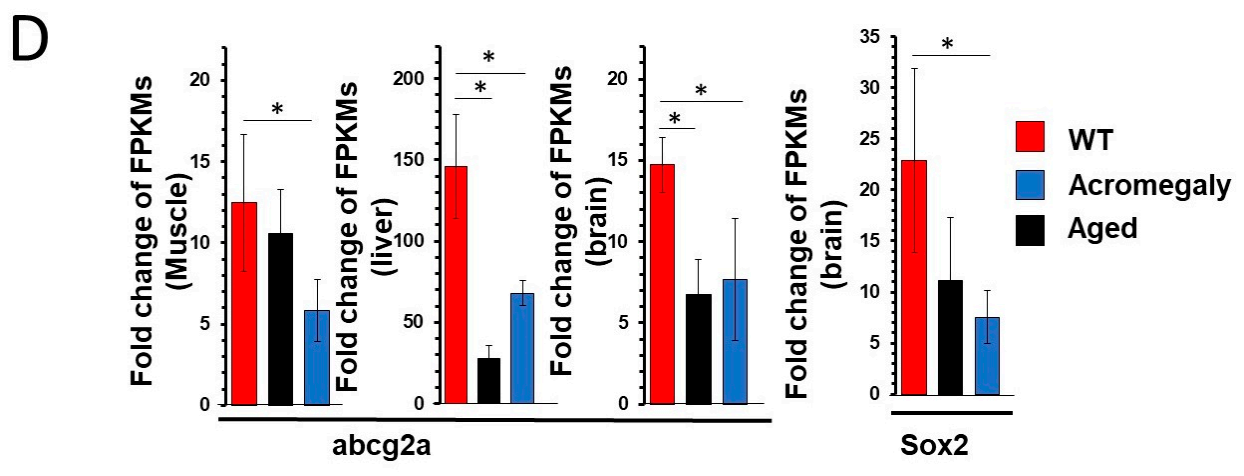
Acromegaly model decline in body stem cell number in various organs. (**A**,**C**) Flow cytometry analysis and quantification of Hoechst 33342 low side population (SP) stem cells as a percent of the total number of cells from the muscle, kidney, and brains of Wild type (WT), acromegaly model (1-year-old), and aged zebrafish (3.5 years old). (**B**,**D**) Stem cell markers abcg2a and Sox2 expression levels in the indicated organs of the WT, acromegaly model, and aged zebrafish (*n* = 3).

As expected, using verapamil at a final concentration of 500 μmol/L with the Hoechst 33342 significantly reduced the SP phenotype in various organs (Figure 1A), indicating that the isolated cells were SP stem cells.

Intriguingly, the proportion of kidney SP stem cells in the acromegaly model was progressively lower than that seen in the wild-type zebrafish, indicating a reduction in the self-renewal ability (Figure 1A). To compare the number of body stem cells between the acromegaly (1-year-old) and aged zebrafish, we identified the SP stem cell population in aged zebrafish (3-year-old) as well. Notably, the decline in the acromegaly kidney SP stem cells was close to that of the aged zebrafish fish (Figure 1A), indicating the drastic effect of excess GH on HSCs cells.

The SP phenotype relies principally on abcg2 gene expression, which has the ability to efflux the Hoechst stain [21]. We performed an RNA-seq data analysis of the mRNA isolated from various organs of the acromegaly (1-year-old), Wild type (WT) (1-year-old), and aged zebrafish (3-year-old).

Likewise, the RNA-seq data analysis showed a significant reduction in the expression of abcg2a in the kidney of the acromegaly zebrafish (Figure 1B).

To investigate whether excess GH reduces SP cells in other organs, we quantified the SP cells in the muscle and brain of the acromegaly zebrafish and matched them with those of the aged zebrafish, as we mentioned earlier. Likewise, excess GH, verapamil treatment, and aging progressively declined the SP cell numbers in the muscle and brain (Figure 1C).

Importantly, the decline in the acromegaly SP cells from various organs was even more apparent than in aged fish (Figure 1C).

Similar to the impact of the excess GH on abcg2a gene expression in the kidney, the RNA-seq data revealed a significant reduction in the expression of abcg2a in various organs, as well as Sox2 (a neural stem cell marker) in the brain of the acromegaly zebrafish (Figure 1D).

**Figure 2:**
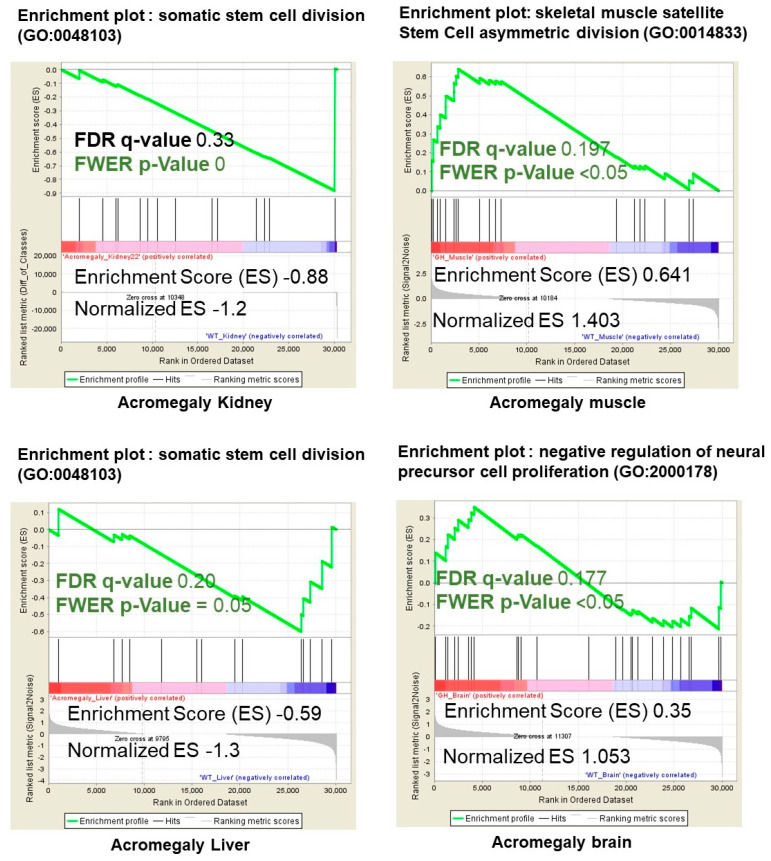
Illustration of statistically significant GSEA results of pathways contributing to stem cell loss and replicative ability in the acromegaly kidney, muscle, liver, and brain. Significant *p*-value < 0.05 and FDR *q*-value < 0.25 are written in red. The reported *p*-value of 0.0 indicates an actual *p*-value of less than 0.01 (*n* = 3).

Consistent with the FACS analysis, the gene set enrichment analysis (GSEA) revealed a significant correlation with pathways contributing to stem cell loss and dysfunction in acromegaly kidney, muscle, liver, and brain samples (Figure 2).

### 2.2. The Acromegaly Model Zebrafish Showing Aging Signs

**Figure 3:**
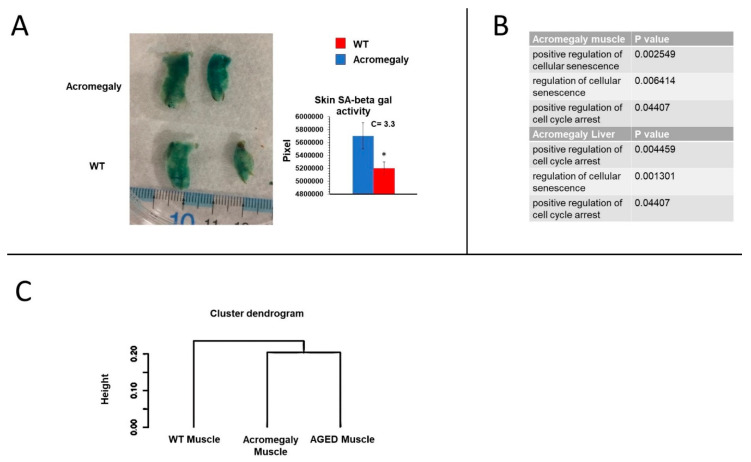
Acromegaly model zebrafish showing aging signs. (**A**) Representative senescence-associated (SA)-β-Gal staining and quantification of WT and acromegaly abdominal skin. Quantification was performed using Fiji software. (**B**) Gene ontology (GO) analysis showed the enrichment of cellular senescence in acromegaly muscle and liver. (**C**) Hierarchical cluster analysis dendrogram of RNA-seq data from muscle of WT, acromegaly model (1-year-old), and aged fish (3-year-old). The *Y*-axis defines the distance between the clusters. Statistical differences (*t*-test, *p* < 0.05) are denoted by asterisks. Data are expressed as the mean ± SE (*n* = 3).

Since the excess GH reduced stem cell numbers in various organs, we studied aging in our model. The zebrafish acromegaly model showed an induction of tissue senescence, demonstrated by the increase in the senescence-associated (SA)-β-galactosidase staining of abdominal skin (Figure 3A). We measured SA-β-gal on the skin because dermal staining showed a strong age association [22].

Moreover, gene ontology (GO) analysis of the RNA-seq data demonstrated the enrichment of cellular senescence and cell cycle arrest in the muscle and liver (Figure 3B). Furthermore, the hierarchical clustering of differentially expressed genes (DEGs) of the muscle RNA-seq data showed similarities between the acromegaly model (1-year-old) and aged (3-year-old) WT zebrafish (Figure 3C). Taken together, these results reinforce the notion that excess GH is associated with aging, rather than longevity. The reduction in stem cell numbers in acromegaly may explain, at least in part, the observed aging signs.

### 2.3. The Acromegaly Model Elevates Oxidative Stress in Various Organs, Including Body Stem Cells

Trying to investigate the possible causes of the reduction of stem cell numbers due to excess GH, we studied oxidative stress in acromegaly.

**Figure 4:**
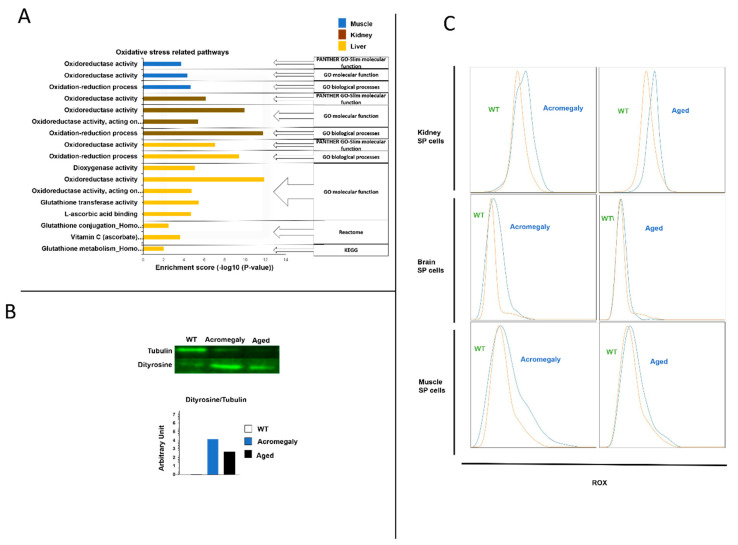
Acromegaly model elevates oxidative stress in various organs including body stem cells. (**A**) The histogram of oxidative stress enriched pathways of DEGs from RNA-seq data in the acromegaly model muscle, kidney, and liver. *Y*-axis, pathway categories; *X*-axis, the statistical significance of the enrichment. (**B**) Dityrosine level in the muscle as measured by Western blot (tubulin was used as the loading control). (**C**) Representative fluorescence-activated cell sorting (FACS) plots showing the oxidative stress level in WT, acromegaly and aged SP stem cells measured by CellROX™ Deep Red staining kits (*n* = 3).

Oxidative stress plays a vital role in stem cell self-renewal. It induces the loss of stem cell self-renewal, the exit of satellite [10], HSCs [11], and neural stem cells [12] from quiescence and the induction of differentiation. To address whetherexcess GH induces oxidative stress in the body and stem cells, we first analyzed the pathway data analysis and GO datasets of DEGs from different organs. We found that the acromegaly model showed an enrichment of oxidative stress-related pathways in various organs (Figure 4A). This finding was confirmed by the Western blotting results from the muscle of the acromegaly model using a dityrosine (an oxidative stress marker) antibody (Figure 4B). As we can see in Figure 4B, the concentration of tubulin in the WT samples was higher. This was because we detected a higher protein concentration in the WT samples measured by the Qubit 2.0 Fluorometer, as described in the Materials and Methods section.

To study the oxidative stress of the acromegaly stem cells, we performed a flow cytometry analysis of the SP cells following CellROX™ Deep Red staining. The data showed a progressive induction of oxidative stress (Figure 4C).

## 3. Discussion

The production of the acromegaly model allowed us to identify some perturbed biological parameters in the acromegaly somatic cells [15] and stem cells. In addition to the induction of DNA damage in somatic cells, here we show that excess GH drastically reduces the number of stem cells in acromegaly.

Patients with acromegaly exhibit some aged related health complications, such as diabetes mellitus, reduced cognitive ability of the brain, and kidney complications [14]. According to this study, these health complications could be due, at least in part, to the induction of aging by excess GH.

The oxidative stress theory of aging is one of the most accepted and studied hypotheses regarding the molecular basis of aging. Over the last five decades, numerous reports have examined the connections between oxidative stress, longevity, and age-related diseases [23]. Oxidative stress has recently been identified as one of the key contributors to stem cell dysfunction [10,11,12].

Oxidative stress induction in acromegaly stem cells might provide a reasonable explanation for the reduction in the number of stem cells in acromegaly. Nevertheless, more research is needed to investigate this point in detail.

Intriguingly, the integrity of the stem cells was affected and the acromegaly zebrafish model showed an increase in tissue senescence, reinforcing the notion that acromegaly patients exhibit premature aging signs [6].

The induction of oxidative stress and tissue senescence are not the only pathways disrupted by excess GH. In our previous publication, we showed that acromegaly zebrafish had increased DNA damage in the somatic cells of various organs [15]. It is not clear, however, whether excess GH increases the DNA damage in stem cells.

Indeed, surface markers are the ideal method for the isolation of stem cells. However, as we know, the surface markers of zebrafish stem cells have not yet been identified, therefore it is appropriate to isolate the SP stem cells using the Hoechst 33342 dye in zebrafish.

SP phenotype and abcg2a gene expression have frequently been used to identify adult stem cells in various human and mouse organs [16,17], but in zebrafish, they have only been described in zebrafish HSCs [18]. In this study, the results of our flow cytometry showed that verapamil treatment progressively decreased the SP cells not only in the kidney but also in the muscle and brain of the zebrafish, indicating that ABC transporters in the muscle and brain are similarly inhibited by verapamil. Importantly, aging, similar to the kidney, has reduced gene expression of abcg2a in other organs. Taken together, these data suggest that the brain and muscle have an SP phenotype.

The whole body staining of SA-β-gal is usually used to detect senescence in adult zebrafish at the tissue level [24]. According to Kishi et al., however, only adult zebrafish skin and dermis were stained with SA-β-gal in their experiment, and dermal staining showed a good correlation with age. Since we did not have enough acromegaly zebrafish necessary for the experiments in that study, we used only the skin for the SA-β-gal, instead of all of the fish. In addition, as we know, SA-β-gal staining has not been used in other zebrafish organs at the tissue level besides whole body staining and zebrafish embryo [24,25].

Although excess GH can induce an increase in muscle mass, it was not associated with an improvement in muscle strength [6]. Conversely, we found a similarity of gene expression pattern between the acromegaly model and aged fish, which is probably due to the decline in satellite cell functionality and number. Taken together, these results suggest that we need to reconsider the value of GH administration in longevity.

## 4. Conclusions

Mutations of the somatotropic axis have been linked with mice and human longevity. The inherited GH receptor mutation significantly extended the life span by over 60% [4]. Here, we show that the overexpression of GH induces premature aging signs in zebrafish, which may be at least partly due to the decline in stem cells.

Laron dwarf mice with GH receptor deficiency have a higher number of bone marrow stem cells compared to their wild siblings [26]. In this study, we showed a decrease in the number of stem cells due to excess GH.

To the best of our knowledge, this is the first study to reveal a reduction in the number of stem cells and an increase in oxidative stress in the stem cells in the acromegaly model.

## 5. Methods

### 5.1. RNA-Seq and Data Analysis

#### 5.1.1. RNA Isolation and Library Construction

All of the experimental procedures were approved by the Tokyo University Animal Care and Ethics Committee. Ethical code: P14-952. The total RNAs were isolated from the muscle, kidney, liver, and brain of adult zebrafish acromegaly model, WT (1-year-old), and aged zebrafish (3 years old), then purified using Trizol reagents (Invitrogen, Carlsbad, CA, USA) according to the manufacturer’s instructions. The total RNAs were purified from three samples of each group. The quantity of RNA was measured by a Qubit RNA Assay Kit using a Qubit 2.0 Fluorometer (Life Technologies, Carlsbad, CA, USA), and the RNA quality was assessed using a 2100 Bioanalyzer (Agilent Technologies, Santa Clara, CA, USA). The cDNA library for Illumina sequencing was prepared according to the instruction of TruSeq standard mRNA Sample Prep Kit (Illumina, San Diego, CA, USA). The RNA sequencing was performed on an Illumina HiSeq 2500 platform (Illumina, San Diego, CA, USA).

#### 5.1.2. Mapping and Differential Gene Expression Calling

HISAT2 software was used according to the instructions from the program designers to map the raw reads of the three-acromegaly zebrafish and WT samples to the zebrafish genome reference (GRCz10) retrieved from the Ensembl database together with the annotation file. The Cufflinks pipeline was used to assign reads to known transcripts and to test for the differential expression between the acromegaly model and the WT samples. The relative transcript abundance was measured in fragments of reads per kilobase of exon sequence per million mapped sequence reads (FPKM).

DEGs (differentially expressed genes) were detected using the cummerbund r package of the cuffdiff file (alpha = adj *p* value < 0.05).

### 5.2. Data Analysis

Gene ontology (GO) (http://geneontology.org/) was used to find enriched biological themes in the acromegaly model zebrafish compared to the WT zebrafish.

The analysis software ENRICHR (http://amp.pharm.mssm.edu/Enrichr/) was used to detect the enriched signaling pathways after converting the differentially expressed genes to their human orthologues using BioMart [27].

Selected GO and ENRICHR pathways were considered significant according to *p* value < 0.05

### 5.3. Gene Set Enrichment Analysis (GSEA)

Using our RNA-seq data, we performed the GSEA [28] analysis by first converting the differentially expressed genes to their human orthologues using Biomart and testing for enrichment against the skeletal muscle satellite stem cell asymmetric division (GO:0014833), the negative regulation of neural precursor cell proliferation (GO:2000178), and somatic stem cell division (GO:0048103) gene sets.

Gene sets were considered significant according to *p* value < 0.05.

### 5.4. Western Blot Analysis

Muscle samples from the acromegaly model, WT (1-year-old), and aged zebrafish (3-year-old) were incubated with a sample buffer (2x Laemmli Sample Buffer, 5% β-mercaptoethanol) and heated directly at 95 °C for 5 min. Protein concentration was measured by the Qubit protein Assay Kit using a Qubit 2.0 Fluorometer (Life Technologies, Carlsbad, CA, USA). The samples containing the protein were loaded onto a 15% SDS-polyacrylamide gel. The proteins were transferred to Polyvinylidene fluoride (PVDF) membranes using the Trans-Blot^®^ Turbo™ Blotting System (Bio-Rad, Hercules, CA, USA) according to the manufacturer’s instructions. The PVDF membranes were incubated in a blocking solution with 5% bovine serum albumin (BSA) in a Tris-buffered saline containing 0.1% Tween20 (TBST) for approximately 1 h at room temperature, with shaking prior to the overnight incubation with mice mAb Dityrosine (SKU, cat. No. #SMC-520), and α Tubulin mice (Santa Cruz Biotechnology, Dallas, TX, cat. No. sc-8035) at a 1/1000 dilution in TBST. The membrane was washed three times for 15 min in TBST before incubation for 1 h at room temperature with a 1/10,000 dilution of Goat anti-Mouse, Alexa Fluor 488 (Abcam, Cambridge, UK, cat. No. ab150113). The membrane was then washed another time with TBST and visualized using the Odyssey^®^ Fc Imaging System (LI-COR, Lincoln, NE, USA).

#### 5.4.1. Body Stem Cell Isolation by FACS

The acromegaly model, WT (1-year-old), and aged zebrafish (3-year-old) were euthanized in ice-cold water for 2 min, then immersed in 70% (vol/vol) ethanol for skin sterilization. The dissection of the zebrafish kidneys was conducted as described previously [29]. Briefly, the collected kidneys in 400 µL of fetal bovine serum (FBS) from 3 fish of each group were disaggregated by repeated trituration with a 1000-µL tip and filtered through the 40-µm EASYstrainer (VWR) for cell isolation. Erythrocytes were depleted by incubation for 2 min with 2 mL of sterile distilled water. Followed by the addition of 4 volumes of hanks’ balanced salt solution (HBSS)/2% FBS, the samples were then washed and resuspended at a concentration of 10^6^/mL in phosphate buffered saline (PBS)/30% FBS. The whole brain was dissected similarly, with the sole exception of omitting the distilled water incubation step. Muscle dissection was conducted as described previously [30] with some modifications. First, 200 mg of muscle from each fish was mechanically disaggregated and incubated in dulbecco’s modified eagle’s medium (DMEM) medium containing 0.2% collagenase (Worthington, Columbus, OH, USA; LS004196) at room temperature with agitation for 60 min, followed by incubation with 0.006% trypsin at room temperature with agitation for 20 min. Cells were filtered through the 40-µm EASYstrainer, then washed and resuspended at a concentration of 10^6^/mL in PBS/30% FBS. Hoechst 33342 dye was added to the kidney, brain, and muscle cell suspension at a final concentration of 3 μg/mL. The mixture was incubated for 90 min at 29 °C. As a negative control, verapamil (Sigma, St. Louis, MO, USA; V4629-1), an inhibitor of ABC transporters, was also added to a final concentration of 500 μmol/L with Hoechst 33342. For oxidative stress detection, CellROX™ Deep Red Reagent (Invitrogen, Carlsbad, CA, USA; C10422) was added to a final concentration of 5 μmol/L and incubated for 90 min at 29 °C. After Hoechst 33342 staining, the cell suspensions were washed by centrifugation, resuspended at 1 × 10^7^ cells/mL in PBS/30% FBS containing 2 μg/mL propidium iodide, and kept on ice until analysis. The FACS analysis was performed using a FACS Aria II (Becton Dickinson, Franklin Lakes, NJ, USA). The Hoechst dye was excited by a 355-nm ultraviolet laser, and its fluorescence was measured at 2 wavelengths using a 424/44 (Hoechst blue) bandpass filter and a 585/42 (Hoechst red) bandpass filter. The CellROX^®^ Deep Red ROS detection reagent was excited by the 639-nm ultraviolet laser, and a 665/40 BP filter was used for the collection of the fluorescence emission. Propidium iodide fluorescence was excited by the 488-nm laser and detected using a 630/22 bandpass filter. Propidium iodide–positive dead cells were excluded. For the flow cytometry analysis and determination of SP cell percentage in each group, we used Cytobank (www.cytobank.org).

#### 5.4.2. SA-b-gal Assay and Quantitation

The abdominal skin of the acromegaly model and the WT zebrafish (1-year-old) were fixed in PBS/4% PFA for 3 days at 4 °C, then washed with PBS-pH 7.4 for 3 × 1 h. They were washed again for 1 h in PBS-pH 6.0 at 4 °C. The staining was performed using a Senescence β-Galactosidase Staining Kit (Cell Signaling #9860) overnight at 37 °C according to the manufacturer’s instructions. SA-β-Gal activity was quantified using the color threshold selection tool in Fiji software.

### 5.5. Statistical Analysis

Unless otherwise indicated, all of the experiments were performed on biological replicates. The sample size is reported in the appropriate figure legends and methods. The results are shown as the average ± s.d. The data were compared for significance using a two-tailed unpaired Student’s *t*-test and deemed statistically significant if the *p*-value was < 0.05.

## Data Availability

The RNA-seq data were submitted to Gene expression omnibus accession number GSE113169.

## Supplementary Materials

The following are available online at https://www.mdpi.com/2079-7737/9/6/120/s1, Figure S1: Flow cytometry gating strategy for isolation of SP population from zebrafish kidney. PE-Texas-Red A/FSC for detection of Propidium iodide–positive dead cells.
