# Supplementary material for: An Acromegaly Disease Zebrafish Model Reveals Decline in Body Stem Cell Number along with Signs of Premature Aging
Source: Biology (Basel). 2020 Jun 7;9(6):120. doi: 10.3390/biology9060120 (PMC7344990; doi:10.3390/biology9060120)
Supplement: Supplementary file 1 [file biology-09-00120-s001.pdf]

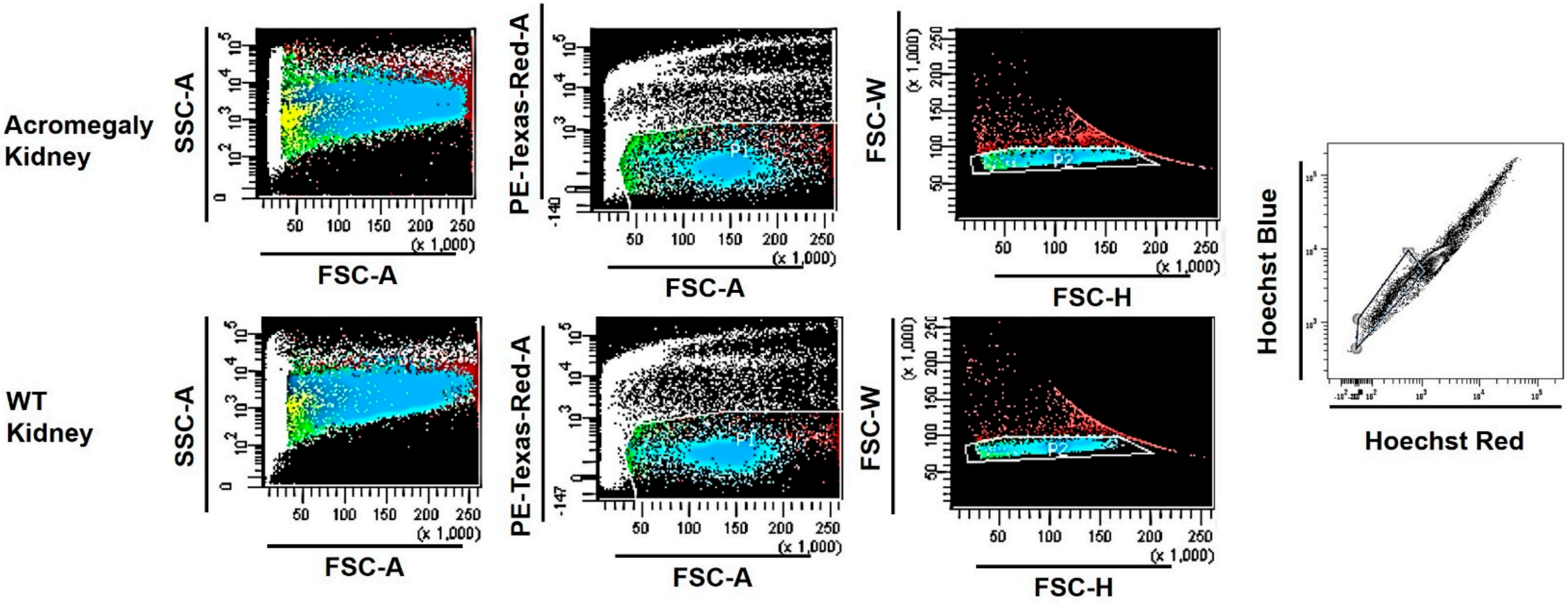

Figure S1. Flow cytometry gating strategy for isolation of SP population from zebrafish kidney. PE-Texas-Red A/FSC for detection of Propidium iodide–positive dead cells.
